# Ca^2+^ Signaling by TRPV4 Channels in Respiratory Function and Disease

**DOI:** 10.3390/cells10040822

**Published:** 2021-04-06

**Authors:** Suhasini Rajan, Christian Schremmer, Jonas Weber, Philipp Alt, Fabienne Geiger, Alexander Dietrich

**Affiliations:** Experimental Pharmacotherapy, Walther-Straub-Institute of Pharmacology and Toxicology, Member of the German Center for Lung Research (DZL), LMU-Munich, 80336 Munich, Germany; suhasini.rajan@lrz.uni-muenchen.de (S.R.); christian.schremmer@lrz.uni-muenchen.de (C.S.); jonweb@gmx.de (J.W.); Philipp.Alt@lrz.uni-muenchen.de (P.A.); Fabienne.Geiger@lrz.uni-muenchen.de (F.G.)

**Keywords:** TRP channels, TRPV4, respiratory system, lung, endothelium, epithelium, Ca^2+^ signaling, signal transduction

## Abstract

Members of the transient receptor potential (TRP) superfamily are broadly expressed in our body and contribute to multiple cellular functions. Most interestingly, the fourth member of the vanilloid family of TRP channels (TRPV4) serves different partially antagonistic functions in the respiratory system. This review highlights the role of TRPV4 channels in lung fibroblasts, the lung endothelium, as well as the alveolar and bronchial epithelium, during physiological and pathophysiological mechanisms. Data available from animal models and human tissues confirm the importance of this ion channel in cellular signal transduction complexes with Ca^2+^ ions as a second messenger. Moreover, TRPV4 is an excellent therapeutic target with numerous specific compounds regulating its activity in diseases, like asthma, lung fibrosis, edema, and infections.

## 1. Introduction

Cation selective ion channels of the transient receptor potential (TRP) superfamily contribute to numerous cellular functions in the body [1]. Originally cloned in *Drosophila* [2,3], we now know 28 proteins in mammals subdivided into six major families: TRPA for ankyrin, TRPC for canonical, TRPM for melastatin, TRPML for mucolipidin, TRPP for polycystin, and TRPV for vanilloid [4,5]. The first cloned member of the latter family (TRPV1), containing six proteins, was identified as vanilloid receptor, which is activated by capsaicin from hot chili peppers [6]. Its fourth protein (TRPV4) was discovered in the year 2000 and is an osmo-sensitive channel homologue [7,8] of the invertebrate gene Osm-9 in *Caenorhabditis elegans*. TRPV4 is functionally expressed almost ubiquitously in human tissues (see Reference [9] for a recent review), and numerous mutations in the protein are linked to diseases, such as skeletal dysplasia and neuropathies (see Reference [10] for a recent review). Here, we review TRPV4 channel expression in the respiratory tract and its function in health and disease.

## 2. Structure and Cellular Function of TRPV4 Channels Modulated by Specific Activators and Inhibitors

**Figure 1:**
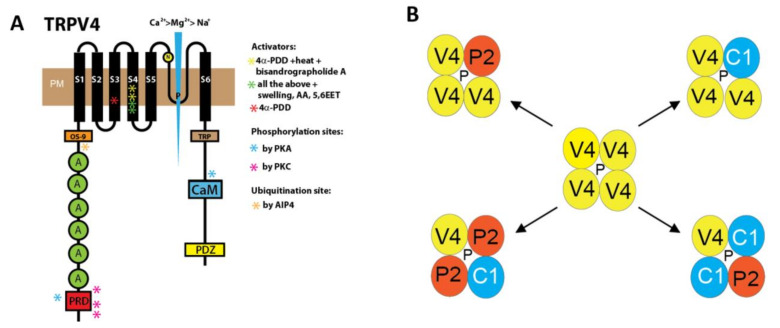
(**A**) Cartoon representing the structure of a he fourth member of the vanilloid family of transient receptor potential channels (TRPV4) channel with functional domains, as well as activator interaction sites (modified from Reference [31]). See text for details. 4α-PDD, 4α-phorbol 12,13 didecanoate; 5,6 EET, 5,6 epoxyeicosatrienoic acids; A, ankyrin repeat domain (ARD); AA, arachidonic acid; CaM, Ca^2+^/calmodulin binding site; N, N-glycosylation site; P, channel pore; PKA, protein kinase A; PKC, protein kinase C; PM, plasma membrane; PDZ, PDZ binding domain; PRD, proline-rich domain; TRP, TRP-box; S1-6, transmembrane segments 1–6. (**B**) A selection of the proposed multimerization potential of TRPV4 (V4) proteins to form functional homo- or together with Transient Receptor Potential Classical 1 (TRPC1) (C1) and Transient Receptor Potential Polycystin 2 (TRPP2) (P2) monomers hetero-tetrameric channels (modified from Reference [32]). P, channel pore.

TRPV4 channels have different functional domains, including a proline-rich domain, six ankyrin repeats domains (ARDs), and an OS-9 binding domain in the amino-terminus. Activating compounds mostly bind to amino acids in transmembrane segments (S) 3 and 4, while the channel pore is located between S5 and S6. The characteristic TRP box, together with a calmodulin (CaM) binding site and a PDZ-binding-like motif are located in the carboxy-terminus of the protein (see Figure 1). While the exact function of the proline-rich domain is still elusive, ARDs are important for trafficking the channel to the plasma membrane, as splice variants with deletions in these repeats show impaired oligomerization and intracellular retention [11]. OS-9 is a ubiquitously expressed endoplasmic reticulum (ER) associated protein which binds to the amino-terminus of TRPV4 channels and aids in TRPV4 maturation [12], while a glycosylation site at N651 [13] is important for membrane targeting (see Figure 1). In addition to homo-oligomers, hetero-tetramers with TRPC1 [14], TRPP2 [15], and both channels [16] were identified (see Figure 1), but it is not known if they are functionally active in native cells. Several phosphorylation sites are located on the amino-terminus (S162, T175, S189, phosphorylated by protein kinase C (PKC) and S184 phosphorylated by protein kinase A (PKA)). On the carboxy-terminus, only one site was identified (S824 for phosphorylation by PKA). All of them sensitize the channel [17,18]. The carboxy-terminal site is also phosphorylated by serum glucocorticoid-induced protein kinase 1 (SGK1) for interaction with F-actin [19] and stromal interaction molecule 1 (STIM1) [20], a sensor protein of the store-operated Ca^2+^ influx [21]. Most interestingly, lung damage by overventilation involves activation of TRPV4 channels phosphorylated at S824 by SGK1 [22]. Ubiquitination of the protein by the ubiquitin ligase AIP4 at the amino-terminus (aa 411–437) promotes endocytosis and decreases channel expression at the plasma membrane [23]. Cryo-electron microscopy (Cryo-EM) and X-ray structures of TRPV4 channels from *Xenopus tropicalis* became available recently and show a wider selectivity filter in the channel pore compared to other TRP channels [24].

TRPV4 channels show a higher Ca^2+^ permeability in comparison to Mg^2+^ and Na^+^ [25,26] and are activated by intracellular Ca^2+^ through binding of Ca^2+^/calmodulin [27]. In the resting state, the amino-terminus forms an auto-inhibitory complex with a carboxy-terminal domain, which is released by Ca^2+^/CaM binding [28]. In clear contrast, other TRP channels, like TRPC3, are inhibited by Ca^2+^/CaM binding [29], while TRPC6 also releases its amino-carboxyl-terminus interaction during activation similar to TRPV4 [30].

TRPV4 was originally described as an osmo-sensing channel, which was activated by hypoosmolar solutions [7,8]. However, the channel pore is also directly opened by cell swelling [33] or mechanical stress (Reference [34], reviewed in Reference [35]) irrespective of other specific triggers. Alternatively, an indirect mechanism by phospholipase A2 (PLA2) and its effectors was described in Müller glia cells [36,37]. While the ARD domains of TRPV4 are not essential for channel opening by cell swelling [7], a stepwise deletion analysis revealed an essential function of the most distal part of the amino-terminus especially the proline-rich domain (PRD) in the activation process [37]. Most interestingly, the cytoskeletal protein PACSIN3 (protein kinase C and casein kinase substrate in neurons protein (3) interacts with this domain in the TRPV4 amino-terminus [38], suggesting changes in the cell cytoskeleton induced by mechanical stress as the ultimate trigger of TRPV4 channel activity [39,40]. Moreover, activation by cell swelling also requires cytochrome P450 (CYP) epo-xygenase activity to convert arachidonic acid (AA) to epoxyeicosatrienoic acids (EETs), such as 5,6-EET and 8,9-EET, which all act as direct TRPV4 agonists in mouse aortic endothelial cells [41]. Reactive oxygen species (ROS) produced by mitochondria also activate TRPV4 channels in endothelial cells of coronary arteries and the systemic vasculature to increase vascular permeability, although the exact molecular mechanism underlying this response is still elusive [42,43]. TRPV4 was described as potential toxicant sensor in many human tissues (reviewed in Reference [44]) as channel inhibition counteracts toxic lung edema in vivo after chlorine exposure [45].

TRPV4 channels are also involved in temperature-sensing from 27 to 44 °C depending on the cell type [7,46], while other TRPV proteins are activated by higher temperatures (TRPV3 > 33°C, TRPV1 > 42 °C and TRPV2 > 52 °C, reviewed in Reference [47]). Moreover, thermosensitivity rises in differences of activation energies associated with voltage dependent opening and closing as described for the closely related TRPV1 channel [48]. The ARDs are essential for activation of TRPV4 by temperature as deletion of the first three proximal domains results in a temperature insensitive channel [49]. The cholesterol content of the plasma membrane seems to be important for TRPV4 channel activation, as supplementation of cholesterol by methyl-β cyclodextrin (MβCD) suppressed temperature-evoked elevations in intracellular Ca^2+^ ([Ca^2+^]_i_) and prolonged the time course of the cell swelling response in TRPV4 expressing Müller cells [50]. Along the same line, phosphatidylinositol-4,5-bisphosphate-dependent rearrangements of the cytosolic tails of TRPV4 are involved in channel activity by both physiological stimuli [51].

A variety of activators for TRPV4 channels have been discovered ranging from natural compounds of plants to synthetic molecules [52]. Bisandrographolide A (BAA) from the plant *Andrographis paniculata*, citric acid, and the flavone apigenin all induce membrane currents in TRPV4-expressing cells [53,54], while phorbolesters, like 4α-phorbol 12,13 didecanoate (4α-PDD), α-phorbol 12,13 dihexanoate (4α-PDH), and phorbol 12-myristate 13-acetate (PMA), open the TRPV4 channel pore [55,56]. For the latter compounds proposed binding sites between S3 and S4 of the channel were identified [57,58]. Most effective is the synthetic lipid GSK1016790A [59,60], which also requires the distant part of the amino-terminus of the channel suggesting a similar activation mechanism as for cell swelling [37]. Recently, local application of quinazolin-4(3 H)-one derivatives as TRPV4 agonists to joint cartilage stimulated chondrocyte matrix production and provided relieve from osteoarthritic damage in a rat model [61].

Inhibitors of TRPV4 channels range from the unspecific compound ruthenium red to more specific blockers, like RN-1734 [62], RN-9893 [63], Crotamin [64], and HC-06704753 [65], with a high therapeutic potential [52]. Most interestingly a hydroxyazetidine TRPV4 inhibitor with a very low half maximal inhibitory concentration (IC_50_) was effective to reverse agonistic effects in rat bladder but failed in other efficacy studies (reviewed in Reference [52]), while another (GSK2798745) is the first TRPV4 antagonist to reach clinical trials [66]. A selection of TRPV4 modulators, their specificity, and their prospective therapeutic options are shown in Table 1.

## 3. TRPV4 Function in the Upper Respiratory Tract and Bronchi

The upper respiratory tract and the trachea are essential to guide inhaled air to the lung for gas exchange but also for protection of the body from inhaled pathogens and toxicants. The pseudostratified epithelium mainly contains goblets cells for the production of mucus and ciliated cells to remove the mucus with invading toxicants and pathogens out of the body. In the bronchi and bronchioles however goblet cells are gradually replaced by club cells, which produce glycosaminoglycans to protect the epithelium (reviewed by Hogan and Tata [70]). By innervation of the upper epithelium sensory afferent nerve impulses are conducted through the vagal nerve to the central nervous system to induce sneezing and coughing [71], which strongly support the removal of pathogens and toxicants.

Activation of TRPV4 channels by specific agonists or hypo-osmotic solution was able to induce depolarization of vagal nerves in humans, mice and guinea pigs, which was blocked by channel antagonists [72]. The authors postulate a signal transduction cascade involving TRPV4, adenosine triphosphate (ATP), and the purinoreceptor 2X3 (P2X3), which is activated by ATP, involved in this sensory airway nerve reflex [72]. Along this line, TRPV4 channels also contributed to the ATP-induced increase in the ciliary beating frequency in ciliated tracheal cells [73]. Importantly, silica nanoparticles, which inhibited TRPV4 activity by GSK1016790A in primary cultured mouse tracheal bronchial epithelial cells, were also able to abrogate this increase in ciliary beating frequency [74]. Moreover, lipopolysaccharides (LPS) released by gram-negative bacteria trigger defensive responses in the airways dependent on Toll-like receptor 4 (TLR-4) via activation of TRPV4 channels [75]. Along this line, TRPV4-deficient mice display exacerbated ventilator changes and recruitment of polymorphonuclear leukocytes into the airways upon LPS challenge [75]

TRPV4 is also important for the regulatory volume decrease (RVD) in airway epithelia. Patients with cystic fibrosis carry mutations in the gene for the cystic fibrosis (CF) transmembrane conductance regulator, an ion channel managing the passage of chloride and bicarbonate ions across the apical membrane of epithelial cells [76]. Most interestingly, RVD is absent in epithelia from CF patients but could be recovered by 4α-phorbolesters as TRPV4 activators [11].

Asthma is a chronic inflammatory disease of the upper airways induced by repeated exposure to specific allergens, which results in activation of epithelial cells and acute bronchoconstriction [77]. In a chicken ovalbumin (OVA) model of asthma, TRPV4-/- mice developed similar levels of airway hyper-responsiveness compared to wild-type (WT) mice [78], although TRPV4 protein levels were increased in WT animals [79]. TRPV4-deficient mice were however protected from airway remodeling in a house dust mite (HDM) model, which is more relevant to the human situation [80]. In nasal cells of patients with allergic rhinitis (AR) caused by HDM, TRPV4 proteins were up-regulated and GSK1016790A as TRPV4 channel activator decreased expression of the cell junctional proteins E-cadherin and Zona occludens 1, which may be responsible for epithelial barrier disruption [81]. TRPV4 agonists induced the release of ATP from human airway smooth muscle cells (HASMC) of non-atopic, immunoglobulin E-independent, asthma patients. ATP as important mediator molecule then activates P2X4 receptors on mast cells to release cysteinyl-leukotriens, which contracted HASMC [82]. Asthma is also triggered by inhalation of allergens from household molds, such as *Aspergillus spec.*, which secrets an alkaline protease Alp1 [83]. After inhalation of Alp1 an immune response involving a T-helper 2 (Th2) cell -induced eosinophilia, cytokine and mucus production, as well as bronchial constriction, occurred [84,85]. Moreover, Alp1 destroys cell junctions of club cells in the airways, which protect the epithelium, detoxify harmful substances and serve as stem cells for ciliated cells [86]. Most interestingly, a club cell specific knock-out of TRPV4 protein resulted in decreased production of the C-C motif chemokine ligand 2 (CCL2) and a reduction of immune cells after inhalation of Alp1 in these mice in comparison to WT controls [87]. Along this line, over-expression of mechanosensitive TRPV4 in these cells resulted in a Ca^2+^/calcineurin-dependent increased Th2 response to Alp1 [87]. Moreover, a single nucleotide polymorphism (SNP) rs6606743 in the human TRPV4 gene increased expression of the channel protein and is associated with fungal immunization and asthma in humans [87]. The same and other TRPV4 related SNPs were also found in patients with chronic obstructive pulmonary disease (COPD) [88]. Importantly, diesel exhaust particles (DEP) evoked protracted Ca^2+^ influx via TRPV4, enhanced by the COPD-predisposing human genetic polymorphism TRPV4P19S [89] Therefore, TRPV4 antagonists may present options for cough relief, asthma, and COPD therapeutics.

## 4. Roles for TRPV4 Channels in Pulmonary Fibroblasts: Key Cells for the Development of Lung Fibrosis

Fibroblasts are involved in repair processes after chronic lung damage by toxicants (e.g., the cytostatic drug bleomycin) and will differentiate to myofibroblasts in response to secreted profibrotic transforming growth factor β (TGF-β1) [90]. Myofibroblasts express α-smooth muscle actin, in addition to secreting large amounts of extracellular matrix, e.g., collagens. They accumulate in fibroproliferative foci, which inhibit gas exchange and may induce lung fibrosis. Despite recent progress with the approval of the drugs pirfenidone and nintedanib for the treatment of pulmonary fibrosis, the only causative treatment is still lung transplantation.

**Figure 2:**
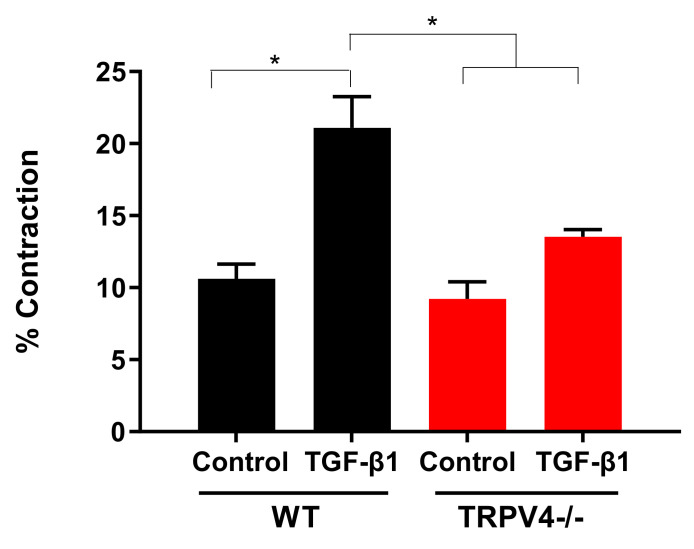
Contraction of primary murine lung fibroblasts (PMLF) in a gel matrix assay (modified from the Doctoral thesis of Jonas Weber (Ludwig-Maximilians-Universität (LMU)-Munich 2020, see http://edoc.ub.uni-muenchen.de/26578/). PMLFs from wild-type (WT) and TRPV4-deficient (TRPV4-/-) mice were seeded in collagen matrices in 6-well plates and transforming growth factor β (TGF-β1) (2 ng/mL, TGF-β1) or solvent (control) was added before release from the well edges (as described in Reference [93]). Diameters of matrices before and after adding TGF-β1/solvent were measured and percent (%) contraction was quantified by calculating differences. Data are shown as means with standard errors of the mean (SEM) of three independent cell isolations (described in Reference [93]). * indicates a *p*-value of <0.05.

TRPV4 is constitutively expressed in primary lung fibroblasts and a global TRPV4-deficient mouse model showed less fibrotic plaques and was partly protected from bleomycin-induced lung fibrosis [91]. Therefore, an additional pathway was proposed by the authors adjunct to TGF-β-signaling. TRPV4-mediated Ca^2+^ influx by mechanical stress induced actomyosin-remodeling and nuclear translocation of myocardin-related transcription factor A (MRTF-A), which activated myofibroblast generation and fibrosis [91]. Along this line, primary murine lung fibroblasts (PMLF) from TRPV4 deficient mice [92] showed less contraction in a collagen gel matrix after adding TGF-β1 in comparison to WT control cells (see Figure 2). Fibroblasts isolated from asthmatic human patients exhibit increased TRPV4 activity compared to cells from healthy donors [80]. The authors propose a TGF-β1-induced signaling pathway via PI3K and TRPV4 to enhance fibrotic gene expression and inhibit fibrinolysis by activating plasminogen activator inhibitor 1 (PAI-1) [80]. Although other cell types, including alveolar epithelial cells (see below), are involved in the development of lung fibrosis [90], TRPV4 antagonists may be useful in pharmacological therapy of lung fibrosis.

## 5. TRPV4 in the Lung Endothelium: Supporting Vasodilation and Lung Permeability

The endothelium provides important pathways for vasodilatation of the systemic and pulmonary circulation (reviewed in Reference [94]). An increase of Ca^2+^ in endothelial cells by stretch-activated TRPV4 channels produces nitric oxide (NO) by the endothelial NO synthetase (eNOS). NO diffuses to the adjacent layer of smooth muscle cells (SMC), stimulates cyclic guanosine monophosphate (cGMP)-signaling activating myosin light chain phosphatase (MLCP), which results in a decrease of contractile force and vasodilation (reviewed in Reference [94]). This mechanism was also dependent on endothelium-derived hyperpolarization factor (EDHF) and caveolar components, as well as connexin proteins at cellular gap junction [95]. Thus, TRPV4 deficient mice were not able to regulate vascular tone and blood pressure, due to lack of channel activity in response to mechanical shear stress [96]. Along this line, activating TRPV4 channels by a higher dose of specific agonist, like GSK1016790A (see Table 1), in mice, rats, or dogs dramatically resulted in a circulatory collapse [68], while, in low doses, the drug produced a decrease in pulmonary arterial pressure in rats [97]. Therefore, TRPV4 activators may serve as new therapeutic option in the fight against pulmonary arterial hypertension (PAH). On the other hand, TRPV4 activation by reactive oxygen species (ROS) in lung microvascular endothelial cells isolated from a rat model of PAH promoted abnormal cell migration and proliferation [98].

The endothelium of the pulmonary circulation is an important cell barrier to protect the lung from toxicants and pathogens circulating in the blood flow, like epithelial cells, in the airways. However, in response to bacterial infections of the lung tissue, endothelial cell permeability increases to facilitate invasion of immune cells from the blood as an essential line of defense. As a side effect of this response, protein-rich fluid accumulates in the lung interstitium and the alveolar space causing an acute and life threatening pulmonary edema (reviewed in Reference [99]). It is assumed that, in analogy to smooth muscle cells, an elevation of the intracellular Ca^2+^ concentration activates myosin light chain kinases (MLCK), promoting actin myosin interaction and triggering a cell shape change of endothelial cells, which results in an increased endothelial permeability (reviewed in Reference [100]).

TRPV4 is a key channel for increasing endothelial permeability (reviewed in Reference [101]; also see Figure 4), and TRPV4-deficiency reduces hydrostatic lung edema formation and capillary leakage [102,103]. Importantly, a TRPV4 antagonist, like GSK2193874 (see Table 1), was effective in inhibiting lung edema by high pulmonary venous pressure, as well as in a myocardial infarction mouse model [69]. Vice versa, activation of TRPV4 by 4α-phorbol esters (see Table 1) initiates lung edema [104]. Two other blockers (GSK2220961, GSK2337429) even protected from acute lung injury, if they were added 30 min after acid aspiration by gastroesophageal reflux [45]. Less effective was GSK2193874, which had to be applied before injury and, therefore, showed only a prophylactic effect [103]. Most interestingly, blocking TRPV4 was also proposed as a promising and feasible approach in the recent coronavirus disease 2019 (COVID-19) pandemic to protect the alveolo-capillary barrier of the lungs, as severe acute respiratory syndrome coronavirus type 2 (SARS-CoV2) is also present in the endothelium [105]. Therefore, the authors suggest GSK2798745 (see Table 1) as a powerful therapeutic option in COVID-19 patients. Unfortunately, in the first clinical trial, LPS-induced elevation of total protein and neutrophils in the bronchoalveolar lavage (BAL) observed after application of this TRPV4 inhibitor from the vascular or the airway site was not different so far in comparison to placebo-treated controls [106]. Therefore, modulating TRPV4 channel activity may be useful in therapeutic approaches for pulmonary hypertension, lung edema, and infection, but clinically successful drugs still need to be established.

## 6. TRPV4 in the Alveolar Epithelium: Reinforcing Lung Barrier Function

**Figure 3:**
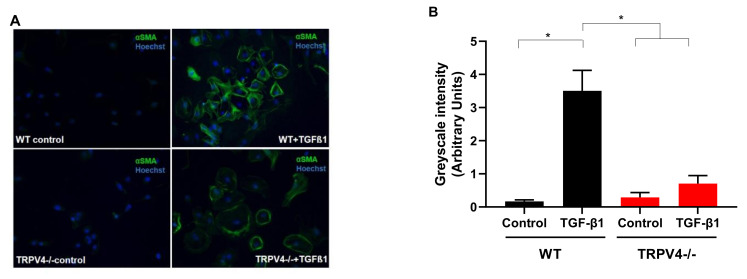
Epithelial mesenchymal transition (EMT) of primary alveolar epithelial cells type 2 (AT2) after application of TGF-β1 quantified by immunostaining of α-smooth muscle actin (α-SMA) and cell nuclei with Hoechst dye (Hoechst). (**A**) Pictures of representative immunohistochemistry stainings of AT2 cells from wild-type (WT) and TRPV4-deficient (TRPV4-/-) mice by a fluorescence-coupled anti-α-SMA antibody (described in Reference [110]) after application of TGF-β1 (+TGF-β1) or solvent (control). (**B**) Summary of data. Grey values of AT2 cells from wild-type (WT) and TRPV4-deficient (TRPV4-/-) mice are plotted. Data are shown as means with standard errors of the mean (SEM) of three independent cell isolations (described in Reference [109]). * indicates a *p*-value of <0.05.

The alveolar epithelium is composed of alveolar type 1 (AT1) and alveolar type 2 (AT2) cells. While AT1 cells with a long and flat shape ensure alveolar epithelial barrier function, cubic AT2 cells produce surfactant to reduce surface tension and enhance gas exchange (reviewed in Reference [70]). In response to severe damage of the alveolar epithelium by pathogens or toxicants leading to cell death and a decrease in barrier function, AT2 cells can differentiate into AT1 cells next to their self-renewal potential. Moreover, in a process called epithelial mesenchymal transition (EMT), AT2 cells transform to mesenchymal cells, which express high amounts of α-smooth muscle actin (α-SMA) and may [107] or may not [108] be involved in wound healing during lung fibrosis. TRPV4 mRNA is expressed in AT2 cells and GSK1016790A (see Table 1) increased basal currents in WT AT2 cells but not in cells from TRPV4-/- mice [109]. While differentiation to AT1 cells was not changed in AT2 cells from TRPV4-deficient mice [109], EMT was significantly reduced in TRPV4-/- AT2 cells compared to WT control cells (Figure 3).

Ischemia-reperfusion (IR)-induced lung injury, which results in alveolar damage, lung edema and hypoxemia remains a significant cause of primary graft failure after lung transplantation [111]. In an isolated perfused mouse lung model, edema formation as one of the hallmarks of IR-induced injury can be mimicked and quantified [112]. Surprisingly, TRPV4-/- lungs showed a significant increase in edema formation compared to WT mice. As these data are in clear contrast to the function of TRPV4 channels in the endothelium (see above), TRPV4 function in the alveolar epithelial barrier was carefully analyzed. Deletion of TRPV4 channels in AT1 cells inhibited aquaporin-5 (AQP-5) expression at the plasma membrane and reduced cell migration, as well as barrier function [109]. Although association of water conducting AQP-5 channels with and regulation by TRPV4 proteins has already been described [113,114], an AQP-5-deficient mouse model showed no differences in the formation of pulmonary edema and iso-osmolar fluid transport from the alveolar space [115]. Importantly, AT2 cells of TRPV4-/- mice showed a decreased production of pro-surfactant protein C, a precursor of surfactant protein-C (SP-C), secreted from these cells and older mice exhibited emphysema-like changes in their lung structure [109]. Next to facilitating gas exchange, surfactant is also important for protection of the alveolar epithelium from chronic micro-injuries. As SP-C-deficient mice show emphysema-like changes [116] similar to TRPV4-/- mice, this phenotype may rather be responsible for the higher edema formation. Importantly, TRPV4 seems to be involved in the protection and regeneration of the alveolar epithelium in older mice, as Ca^2+^ influx through TRPV4 channels are important for production of surfactant, as well as barrier function by AT1 cells.

**Figure 4:**
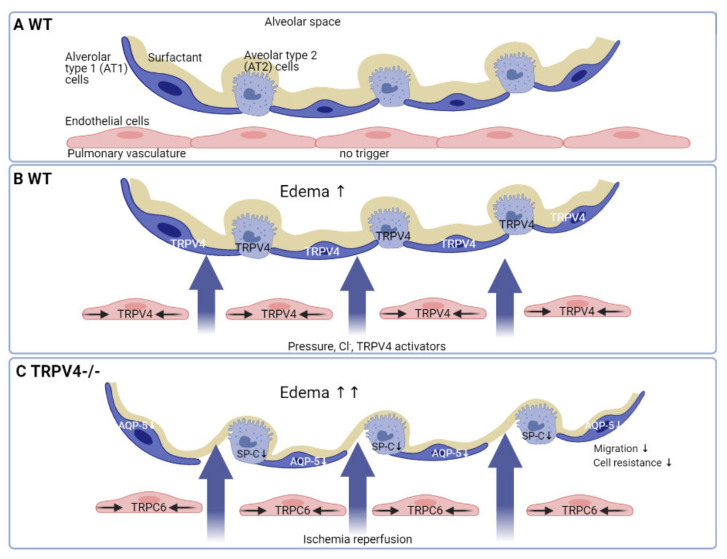
A model for edema formation by different triggers in wild-type (WT) and TRPV4-deficient (TRPV4-/-) lungs. (**A**) The alveolar capillary membrane with resident cells in the quiescent state (no trigger) in wild-type (WT) mice. (**B**) TRPV4 channels are expressed in endothelial, AT1 and AT2 cells. Edema formation by pressure, Cl^-^ and other TRPV4 activators in WT lungs is due to increased endothelial permeability by TRPV4 channels (reviewed in Reference [101]). (**C**) Stronger edema formation by ischemia-reperfusion (IR) in TRPV4-deficient (TRPV4-/-) mice. IR-induced edema is dependent on acute activation of TRPC6 channels in the vascular endothelium and supported by a chronic loss of barrier function in the alveolar epithelium due to ablation of TRPV4 channels. TRPV4 deficiency results in reduced surfactant protein-C (SP-C) production in AT2 and decreased aquaporin-5 (AQP-5) expression in AT1 cells, which also showed less barrier function and reduced cell migration [109].

All these data support a model of increased edema formation by endothelial TRPV4 channels induced by mechanical ventilation and application of toxicants (see above), while deletion of TRPV4 proteins in the alveolar epithelium and activation of TRPC6 channels in the endothelium results in significantly higher edema formation by IR (see Figure 4). Most interestingly, edema formation by these chronic changes in the TRPV4-/- epithelium are partly reversed in TRPC6/TRPV4-double deficient lungs [109], as TRPC6 ablation reduces IR-induced lung edema formation by a loss of permeability in the pulmonary endothelium [117]. Therefore, TRPV4 serves different partly antagonistic function in the formation of lung edema dependent on the tissue, where the channel is expressed (Figure 4). This interesting phenomenon needs to be further analyzed in time- and tissue-specific TRPV4-deficient mouse models, as well as human tissues, in the near future.

## 7. Conclusions

In summary, TRPV4 proteins are expressed in numerous cells of the respiratory tract, like ciliated, club, AT1, and AT2 cells of the airways, as well as endothelial cells of the pulmonary vasculature. Channel function is essential for a first line of defense against invading pathogens and toxicants, while channel dysfunction is coupled to diseases, like asthma, COPD, CF, lung fibrosis, and edema. Although many specific TRPV4 modulators are available, none of them has succeeded in clinical trials so far. Nevertheless, TRPV4 channels are important pharmacological targets as new therapeutic option for respiratory disease.

## Figures and Tables

**Table 1:** Selected compounds activating or inhibiting TRPV4 channels (modified from Reference [67]).

Drug	TRPV4	TRPV1	TRPV2/3	IC_50_/EC_50_^*^	Therapeutic opt.	Ref.
α-phorbol-e.	+	/	/	0.37 µM	-	[56]
EETs	+	?	?	0.15 µM	-	[41]
GSK1016790A	+	/	?	5 nM	Blood pressure↓	[59,68]
RN1747	+	?	?	0.77 µM	-	[62]
Quinazolin d.	+	?	?	60 nM	Osteoarthritis	[61]
RN1734	-	/	/	2.3 µM	-	[62]
HC-06704753	-	/	/	48 nM	Cystitis, COPD	[65]
GSK2193874	-	?	?	40 nM	Pulm. Edema	[69]
RN9893	-	?	?	0.42 µM		[63]
PF-0514030	-	?	?	4 nM	Bladder activity↓	[52]
GSK2798745	-	?	?	1.8 nM	Phase I studies	[66]

*, modified from Reference [52]; +, activating; d., derivate; e., esters; -, inhibiting; ?, not tested; /, very low activity; ↓, reduction, opt., options, Ref., reference.

## Data Availability

Data sharing is not applicable to this article.

## References

[1] Nilius B., Szallasi A. (2014). Transient receptor potential channels as drug targets: From the science of basic research to the art of medicine. Pharmacol. Rev..

[2] Montell C., Rubin G.M. (1989). Molecular characterization of the Drosophila trp locus: A putative integral membrane protein required for phototransduction. Neuron.

[3] Hardie R.C., Minke B. (1992). The trp gene is essential for a light-activated Ca^2+^ channel in Drosophila photoreceptors. Neuron.

[4] Montell C., Birnbaumer L., Flockerzi V., Bindels R.J., Bruford E.A., Caterina M.J., Clapham D.E., Harteneck C., Heller S., Julius D. (2002). A unified nomenclature for the superfamily of TRP cation channels. Mol. Cell.

[5] Dietrich A. (2019). Transient Receptor Potential (TRP) Channels in Health and Disease. Cells.

[6] Caterina M.J., Schumacher M.A., Tominaga M., Rosen T.A., Levine J.D., Julius D. (1997). The capsaicin receptor: A heat-activated ion channel in the pain pathway. Nature.

[7] Liedtke W., Choe Y., Marti-Renom M.A., Bell A.M., Denis C.S., Sali A., Hudspeth A.J., Friedman J.M., Heller S. (2000). Vanilloid receptor-related osmotically activated channel (VR-OAC), a candidate vertebrate osmoreceptor. Cell.

[8] Strotmann R., Harteneck C., Nunnenmacher K., Schultz G., Plant T.D. (2000). OTRPC4, a nonselective cation channel that confers sensitivity to extracellular osmolarity. Nat. Cell Biol..

[9] Rosenbaum T., Benitez-Angeles M., Sanchez-Hernandez R., Morales-Lazaro S.L., Hiriart M., Morales-Buenrostro L.E., Torres-Quiroz F. (2020). TRPV4: A Physio and Pathophysiologically Significant Ion Channel. Int. J. Mol. Sci..

[10] Toft-Bertelsen T.L., MacAulay N. (2021). TRPing to the Point of Clarity: Understanding the Function of the Complex TRPV4 Ion Channel. Cells.

[11] Arniges M., Fernandez-Fernandez J.M., Albrecht N., Schaefer M., Valverde M.A. (2006). Human TRPV4 channel splice variants revealed a key role of ankyrin domains in multimerization and trafficking. J. Biol. Chem..

[12] Wang Y., Fu X., Gaiser S., Kottgen M., Kramer-Zucker A., Walz G., Wegierski T. (2007). OS-9 regulates the transit and polyubiquitination of TRPV4 in the endoplasmic reticulum. J. Biol. Chem..

[13] Xu H., Fu Y., Tian W., Cohen D.M. (2006). Glycosylation of the osmoresponsive transient receptor potential channel TRPV4 on Asn-651 influences membrane trafficking. Am. J. Physiol. Renal Physiol..

[14] Ma X., Nilius B., Wong J.W., Huang Y., Yao X. (2011). Electrophysiological properties of heteromeric TRPV4-C1 channels. Biochim. Biophys. Acta.

[15] Stewart A.P., Smith G.D., Sandford R.N., Edwardson J.M. (2010). Atomic force microscopy reveals the alternating subunit arrangement of the TRPP2-TRPV4 heterotetramer. Biophys. J..

[16] Du W., Huang J., Yao H., Zhou K., Duan B., Wang Y. (2010). Inhibition of TRPC6 degradation suppresses ischemic brain damage in rats. J. Clin. Investig..

[17] Fan H.C., Zhang X., McNaughton P.A. (2009). Activation of the TRPV4 ion channel is enhanced by phosphorylation. J. Biol. Chem..

[18] Peng H., Lewandrowski U., Muller B., Sickmann A., Walz G., Wegierski T. (2010). Identification of a Protein Kinase C-dependent phosphorylation site involved in sensitization of TRPV4 channel. Biochem. Biophys. Res. Commun..

[19] Shin S.H., Lee E.J., Hyun S., Chun J., Kim Y., Kang S.S. (2012). Phosphorylation on the Ser 824 residue of TRPV4 prefers to bind with F-actin than with microtubules to expand the cell surface area. Cell Signal..

[20] Shin S.H., Lee E.J., Chun J., Hyun S., Kang S.S. (2015). Phosphorylation on TRPV4 Serine 824 Regulates Interaction with STIM1. Open Biochem. J..

[21] Cahalan M.D. (2009). STIMulating store-operated Ca(2+) entry. Nat. Cell Biol..

[22] Michalick L., Erfinanda L., Weichelt U., van der Giet M., Liedtke W., Kuebler W.M. (2017). Transient Receptor Potential Vanilloid 4 and Serum Glucocorticoid-regulated Kinase 1 Are Critical Mediators of Lung Injury in Overventilated Mice In Vivo. Anesthesiology.

[23] Wegierski T., Hill K., Schaefer M., Walz G. (2006). The HECT ubiquitin ligase AIP4 regulates the cell surface expression of select TRP channels. EMBO J..

[24] Deng Z., Paknejad N., Maksaev G., Sala-Rabanal M., Nichols C.G., Hite R.K., Yuan P. (2018). Cryo-EM and X-ray structures of TRPV4 reveal insight into ion permeation and gating mechanisms. Nat. Struct. Mol. Biol..

[25] Voets T., Prenen J., Vriens J., Watanabe H., Janssens A., Wissenbach U., Bodding M., Droogmans G., Nilius B. (2002). Molecular determinants of permeation through the cation channel TRPV4. J. Biol. Chem..

[26] Watanabe H., Vriens J., Janssens A., Wondergem R., Droogmans G., Nilius B. (2003). Modulation of TRPV4 gating by intra- and extracellular Ca^2+^. Cell Calcium.

[27] Strotmann R., Schultz G., Plant T.D. (2003). Ca^2+^-dependent potentiation of the nonselective cation channel TRPV4 is mediated by a C-terminal calmodulin binding site. J. Biol. Chem..

[28] Strotmann R., Semtner M., Kepura F., Plant T.D., Schoneberg T. (2010). Interdomain interactions control Ca^2+^-dependent potentiation in the cation channel TRPV4. PLoS ONE.

[29] Zhang Z., Tang J., Tikunova S., Johnson J.D., Chen Z., Qin N., Dietrich A., Stefani E., Birnbaumer L., Zhu M.X. (2001). Activation of Trp3 by inositol 1,4,5-trisphosphate receptors through displacement of inhibitory calmodulin from a common binding domain. Proc. Natl. Acad. Sci. USA.

[30] Fiedler S., Storch U., Erdogmus S., Gudermann T., Mederos Y.S.M., Dietrich A. (2019). Small Fluorescein Arsenical Hairpin-Based Forster Resonance Energy Transfer Analysis Reveals Changes in Amino- to Carboxyl-Terminal Interactions upon OAG Activation of Classical Transient Receptor Potential 6. Mol. Pharmacol..

[31] Dietrich A., Steinritz D., Gudermann T. (2017). Transient receptor potential (TRP) channels as molecular targets in lung toxicology and associated diseases. Cell Calcium.

[32] Dietrich A., Fahlbusch M., Gudermann T. (2014). Classical Transient Receptor Potential 1 (TRPC1): Channel or Channel Regulator?. Cells.

[33] Jo A.O., Ryskamp D.A., Phuong T.T., Verkman A.S., Yarishkin O., MacAulay N., Krizaj D. (2015). TRPV4 and AQP4 Channels Synergistically Regulate Cell Volume and Calcium Homeostasis in Retinal Muller Glia. J. Neurosci..

[34] Gao X., Wu L., O’Neil R.G. (2003). Temperature-modulated diversity of TRPV4 channel gating: Activation by physical stresses and phorbol ester derivatives through protein kinase C-dependent and -independent pathways. J. Biol. Chem..

[35] Michalick L., Kuebler W.M. (2020). TRPV4-A Missing Link Between Mechanosensation and Immunity. Front. Immunol..

[36] Ryskamp D.A., Jo A.O., Frye A.M., Vazquez-Chona F., MacAulay N., Thoreson W.B., Krizaj D. (2014). Swelling and eicosanoid metabolites differentially gate TRPV4 channels in retinal neurons and glia. J. Neurosci..

[37] Toft-Bertelsen T.L., Yarishkin O., Redmon S., Phuong T.T.T., Krizaj D., MacAulay N. (2019). Volume sensing in the transient receptor potential vanilloid 4 ion channel is cell type-specific and mediated by an N-terminal volume-sensing domain. J. Biol. Chem..

[38] D’Hoedt D., Owsianik G., Prenen J., Cuajungco M.P., Grimm C., Heller S., Voets T., Nilius B. (2008). Stimulus-specific modulation of the cation channel TRPV4 by PACSIN 3. J. Biol. Chem..

[39] Ramadass R., Becker D., Jendrach M., Bereiter-Hahn J. (2007). Spectrally and spatially resolved fluorescence lifetime imaging in living cells: TRPV4-microfilament interactions. Arch Biochem. Biophys..

[40] Ji C., McCulloch C.A. (2020). TRPV4 integrates matrix mechanosensing with Ca(2+) signaling to regulate extracellular matrix remodeling. FEBS J..

[41] Vriens J., Owsianik G., Fisslthaler B., Suzuki M., Janssens A., Voets T., Morisseau C., Hammock B.D., Fleming I., Busse R. (2005). Modulation of the Ca^2+^ Permeable Cation Channel TRPV4 by Cytochrome P450 Epoxygenases in Vascular Endothelium. Circ. Res..

[42] Bubolz A.H., Mendoza S.A., Zheng X., Zinkevich N.S., Li R., Gutterman D.D., Zhang D.X. (2012). Activation of endothelial TRPV4 channels mediates flow-induced dilation in human coronary arterioles: Role of Ca^2+^ entry and mitochondrial ROS signaling. Am. J. Physiol. Heart Circ. Physiol..

[43] Suresh K., Servinsky L., Reyes J., Baksh S., Undem C., Caterina M., Pearse D.B., Shimoda L.A. (2015). Hydrogen peroxide-induced calcium influx in lung microvascular endothelial cells involves TRPV4. Am. J. Physiol. Lung Cell. Mol. Physiol..

[44] Steinritz D., Stenger B., Dietrich A., Gudermann T., Popp T. (2018). TRPs in Tox: Involvement of Transient Receptor Potential-Channels in Chemical-Induced Organ Toxicity-A Structured Review. Cells.

[45] Balakrishna S., Song W., Achanta S., Doran S.F., Liu B., Kaelberer M.M., Yu Z., Sui A., Cheung M., Leishman E. (2014). TRPV4 inhibition counteracts edema and inflammation and improves pulmonary function and oxygen saturation in chemically induced acute lung injury. Am. J. Physiol. Lung Cell. Mol. Physiol..

[46] Guler A.D., Lee H., Iida T., Shimizu I., Tominaga M., Caterina M. (2002). Heat-evoked activation of the ion channel, TRPV4. J. Neurosci..

[47] Belvisi M.G., Dubuis E., Birrell M.A. (2011). Transient receptor potential A1 channels: Insights into cough and airway inflammatory disease. Chest.

[48] Voets T., Droogmans G., Wissenbach U., Janssens A., Flockerzi V., Nilius B. (2004). The principle of temperature-dependent gating in cold- and heat-sensitive TRP channels. Nature.

[49] Watanabe H., Vriens J., Suh S.H., Benham C.D., Droogmans G., Nilius B. (2002). Heat-evoked activation of TRPV4 channels in a HEK293 cell expression system and in native mouse aorta endothelial cells. J. Biol. Chem..

[50] Lakk M., Yarishkin O., Baumann J.M., Iuso A., Krizaj D. (2017). Cholesterol regulates polymodal sensory transduction in Muller glia. Glia.

[51] Garcia-Elias A., Mrkonjic S., Pardo-Pastor C., Inada H., Hellmich U.A., Rubio-Moscardo F., Plata C., Gaudet R., Vicente R., Valverde M.A. (2013). Phosphatidylinositol-4,5-biphosphate-dependent rearrangement of TRPV4 cytosolic tails enables channel activation by physiological stimuli. Proc. Natl. Acad. Sci. USA.

[52] Lawhorn B.G., Brnardic E.J., Behm D.J. (2020). Recent advances in TRPV4 agonists and antagonists. Bioorg. Med. Chem. Lett..

[53] Watanabe H., Vriens J., Prenen J., Droogmans G., Voets T., Nilius B. (2003). Anandamide and arachidonic acid use epoxyeicosatrienoic acids to activate TRPV4 channels. Nature.

[54] Smith P.L., Maloney K.N., Pothen R.G., Clardy J., Clapham D.E. (2006). Bisandrographolide from Andrographis paniculata activates TRPV4 channels. J. Biol. Chem..

[55] Watanabe H., Davis J.B., Smart D., Jerman J.C., Smith G.D., Hayes P., Vriens J., Cairns W., Wissenbach U., Prenen J. (2002). Activation of TRPV4 channels (hVRL-2/mTRP12) by phorbol derivatives. J. Biol. Chem..

[56] Klausen T.K., Pagani A., Minassi A., Ech-Chahad A., Prenen J., Owsianik G., Hoffmann E.K., Pedersen S.F., Appendino G., Nilius B. (2009). Modulation of the transient receptor potential vanilloid channel TRPV4 by 4alpha-phorbol esters: A structure-activity study. J. Med. Chem..

[57] Vriens J., Owsianik G., Janssens A., Voets T., Nilius B. (2007). Determinants of 4 alpha-phorbol sensitivity in transmembrane domains 3 and 4 of the cation channel TRPV4. J. Biol. Chem..

[58] Klausen T.K., Janssens A., Prenen J., Owsianik G., Hoffmann E.K., Pedersen S.F., Nilius B. (2014). Single point mutations of aromatic residues in transmembrane helices 5 and -6 differentially affect TRPV4 activation by 4alpha-PDD and hypotonicity: Implications for the role of the pore region in regulating TRPV4 activity. Cell Calcium.

[59] Thorneloe K.S., Sulpizio A.C., Lin Z., Figueroa D.J., Clouse A.K., McCafferty G.P., Chendrimada T.P., Lashinger E.S., Gordon E., Evans L. (2008). N-((1S)-1-{[4-((2S)-2-{[(2,4-dichlorophenyl)sulfonyl]amino}-3-hydroxypropanoyl)-1 -piperazinyl]carbonyl}-3-methylbutyl)-1-benzothiophene-2-carboxamide (GSK1016790A), a novel and potent transient receptor potential vanilloid 4 channel agonist induces urinary bladder contraction and hyperactivity: Part I. J. Pharmacol. Exp. Ther..

[60] Jin M., Wu Z., Chen L., Jaimes J., Collins D., Walters E.T., O’Neil R.G. (2011). Determinants of TRPV4 activity following selective activation by small molecule agonist GSK1016790A. PLoS ONE.

[61] Atobe M., Nagami T., Muramatsu S., Ohno T., Kitagawa M., Suzuki H., Ishiguro M., Watanabe A., Kawanishi M. (2019). Discovery of Novel Transient Receptor Potential Vanilloid 4 (TRPV4) Agonists as Regulators of Chondrogenic Differentiation: Identification of Quinazolin-4(3 H)-ones and in Vivo Studies on a Surgically Induced Rat Model of Osteoarthritis. J. Med. Chem..

[62] Vincent F., Acevedo A., Nguyen M.T., Dourado M., DeFalco J., Gustafson A., Spiro P., Emerling D.E., Kelly M.G., Duncton M.A. (2009). Identification and characterization of novel TRPV4 modulators. Biochem. Biophys. Res. Commun..

[63] Vincent F., Duncton M.A. (2011). TRPV4 agonists and antagonists. Curr. Top. Med. Chem..

[64] Kittaka H., Yamanoi Y., Tominaga M. (2017). Transient receptor potential vanilloid 4 (TRPV4) channel as a target of crotamiton and its bimodal effects. Pflugers Arch..

[65] Everaerts W., Zhen X., Ghosh D., Vriens J., Gevaert T., Gilbert J.P., Hayward N.J., McNamara C.R., Xue F., Moran M.M. (2010). Inhibition of the cation channel TRPV4 improves bladder function in mice and rats with cyclophosphamide-induced cystitis. Proc. Natl. Acad. Sci. USA.

[66] Brooks C.A., Barton L.S., Behm D.J., Brnardic E.J., Costell M.H., Holt D.A., Jolivette L.J., Matthews J.M., McAtee J.J., McCleland B.W. (2019). Discovery of GSK3527497: A Candidate for the Inhibition of Transient Receptor Potential Vanilloid-4 (TRPV4). J. Med. Chem..

[67] Dietrich A. (2019). Modulators of Transient Receptor Potential (TRP) Channels as Therapeutic Options in Lung Disease. Pharmaceuticals.

[68] Willette R.N., Bao W., Nerurkar S., Yue T.L., Doe C.P., Stankus G., Turner G.H., Ju H., Thomas H., Fishman C.E. (2008). Systemic activation of the transient receptor potential vanilloid subtype 4 channel causes endothelial failure and circulatory collapse: Part 2. J. Pharmacol. Exp. Ther..

[69] Thorneloe K.S., Cheung M., Bao W., Alsaid H., Lenhard S., Jian M.Y., Costell M., Maniscalco-Hauk K., Krawiec J.A., Olzinski A. (2012). An orally active TRPV4 channel blocker prevents and resolves pulmonary edema induced by heart failure. Sci. Transl. Med..

[70] Hogan B., Tata P.R. (2019). Cellular organization and biology of the respiratory system. Nat. Cell Biol..

[71] Canning B.J. (2011). Functional implications of the multiple afferent pathways regulating cough. Pulm. Pharmacol. Ther..

[72] Bonvini S.J., Birrell M.A., Grace M.S., Maher S.A., Adcock J.J., Wortley M.A., Dubuis E., Ching Y.M., Ford A.P., Shala F. (2016). Transient receptor potential cation channel, subfamily V, member 4 and airway sensory afferent activation: Role of adenosine triphosphate. J. Allergy Clin. Immunol..

[73] Lorenzo I.M., Liedtke W., Sanderson M.J., Valverde M.A. (2008). TRPV4 channel participates in receptor-operated calcium entry and ciliary beat frequency regulation in mouse airway epithelial cells. Proc. Natl. Acad. Sci. USA.

[74] Sanchez A., Alvarez J.L., Demydenko K., Jung C., Alpizar Y.A., Alvarez-Collazo J., Cokic S.M., Valverde M.A., Hoet P.H., Talavera K. (2017). Silica nanoparticles inhibit the cation channel TRPV4 in airway epithelial cells. Part Fibre Toxicol..

[75] Alpizar Y.A., Boonen B., Sanchez A., Jung C., Lopez-Requena A., Naert R., Steelant B., Luyts K., Plata C., De Vooght V. (2017). TRPV4 activation triggers protective responses to bacterial lipopolysaccharides in airway epithelial cells. Nat. Commun..

[76] Borowitz D. (2015). CFTR, bicarbonate, and the pathophysiology of cystic fibrosis. Pediatr. Pulmonol..

[77] Lemanske R.F., Busse W.W. (2003). 6. Asthma. J. Allergy Clin. Immunol..

[78] Palaniyandi S., Rajendrakumar A.M., Periasamy S., Goswami R., Tuo W., Zhu X., Rahaman S.O. (2020). TRPV4 is dispensable for the development of airway allergic asthma. Lab. Investig. J. Tech. Methods Pathol..

[79] Kim B.G., Park M.K., Lee P.H., Lee S.H., Hong J., Aung M.M.M., Moe K.T., Han N.Y., Jang A.S. (2020). Effects of nanoparticles on neuroinflammation in a mouse model of asthma. Respir. Physiol. Neurobiol..

[80] Gombedza F., Kondeti V., Al-Azzam N., Koppes S., Duah E., Patil P., Hexter M., Phillips D., Thodeti C.K., Paruchuri S. (2017). Mechanosensitive transient receptor potential vanilloid 4 regulates Dermatophagoides farinae-induced airway remodeling via 2 distinct pathways modulating matrix synthesis and degradation. FASEB J..

[81] Lee K., Byun J., Kim B., Yeon J., Tai J., Lee S.H., Kim T.H. (2020). TRPV4-Mediated Epithelial Junction Disruption in Allergic Rhinitis Triggered by House Dust Mites. Am. J. Rhinol. Allergy.

[82] Bonvini S.J., Birrell M.A., Dubuis E., Adcock J.J., Wortley M.A., Flajolet P., Bradding P., Belvisi M.G. (2020). Novel airway smooth muscle-mast cell interactions and a role for the TRPV4-ATP axis in non-atopic asthma. Eur. Respir J..

[83] Wartenberg D., Lapp K., Jacobsen I.D., Dahse H.M., Kniemeyer O., Heinekamp T., Brakhage A.A. (2011). Secretome analysis of Aspergillus fumigatus reveals Asp-hemolysin as a major secreted protein. Int. J. Med. Microbiol..

[84] Lambrecht B.N., Hammad H. (2015). The immunology of asthma. Nat. Immunol..

[85] McKenzie A.N. (2014). Type-2 innate lymphoid cells in asthma and allergy. Ann. Am. Thorac. Soc..

[86] Ciechanowicz A. (2019). Stem Cells in Lungs. Adv. Exp. Med. Biol..

[87] Wiesner D.L., Merkhofer R.M., Ober C., Kujoth G.C., Niu M., Keller N.P., Gern J.E., Brockman-Schneider R.A., Evans M.D., Jackson D.J. (2020). Club Cell TRPV4 Serves as a Damage Sensor Driving Lung Allergic Inflammation. Cell Host Microbe.

[88] Zhu G., Gulsvik A., Bakke P., Ghatta S., Anderson W., Lomas D.A., Silverman E.K., Pillai S.G. (2009). Association of TRPV4 gene polymorphisms with chronic obstructive pulmonary disease. Hum. Mol. Genet..

[89] Li J., Kanju P., Patterson M., Chew W.L., Cho S.H., Gilmour I., Oliver T., Yasuda R., Ghio A., Simon S.A. (2011). TRPV4-mediated calcium influx into human bronchial epithelia upon exposure to diesel exhaust particles. Environ. Health Perspect..

[90] King T.E., Pardo A., Selman M. (2011). Idiopathic pulmonary fibrosis. Lancet.

[91] Rahaman S.O., Grove L.M., Paruchuri S., Southern B.D., Abraham S., Niese K.A., Scheraga R.G., Ghosh S., Thodeti C.K., Zhang D.X. (2014). TRPV4 mediates myofibroblast differentiation and pulmonary fibrosis in mice. J. Clin. Investig..

[92] Suzuki M., Mizuno A., Kodaira K., Imai M. (2003). Impaired pressure sensation in mice lacking TRPV4. J. Biol. Chem..

[93] Hofmann K., Fiedler S., Vierkotten S., Weber J., Klee S., Jia J., Zwickenpflug W., Flockerzi V., Storch U., Yildirim A.O. (2017). Classical transient receptor potential 6 (TRPC6) channels support myofibroblast differentiation and development of experimental pulmonary fibrosis. Biochim. Biophys. Acta.

[94] Chen Y.L., Sonkusare S.K. (2020). Endothelial TRPV4 channels and vasodilator reactivity. Curr. Top. Membr..

[95] Rath G., Saliez J., Behets G., Romero-Perez M., Leon-Gomez E., Bouzin C., Vriens J., Nilius B., Feron O., Dessy C. (2012). Vascular hypoxic preconditioning relies on TRPV4-dependent calcium influx and proper intercellular gap junctions communication. Arterioscler. Thromb. Vasc. Biol..

[96] Hartmannsgruber V., Heyken W.T., Kacik M., Kaistha A., Grgic I., Harteneck C., Liedtke W., Hoyer J., Kohler R. (2007). Arterial response to shear stress critically depends on endothelial TRPV4 expression. PLoS ONE.

[97] Pankey E.A., Zsombok A., Lasker G.F., Kadowitz P.J. (2014). Analysis of responses to the TRPV4 agonist GSK1016790A in the pulmonary vascular bed of the intact-chest rat. Am. J. Physiol. Heart Circ. Physiol..

[98] Suresh K., Servinsky L., Jiang H., Bigham Z., Yun X., Kliment C., Huetsch J., Damarla M., Shimoda L.A. (2018). Reactive oxygen species induced Ca(2+) influx via TRPV4 and microvascular endothelial dysfunction in the SU5416/hypoxia model of pulmonary arterial hypertension. Am. J. Physiol. Lung Cell. Mol. Physiol..

[99] Ware L.B., Matthay M.A. (2005). Clinical practice. Acute pulmonary edema. N. Engl. J. Med..

[100] Gudermann T., Steinritz D. (2013). STIMulating stress fibers in endothelial cells. Sci. Signal..

[101] Simmons S., Erfinanda L., Bartz C., Kuebler W.M. (2018). Novel mechanisms regulating endothelial barrier function in the pulmonary microcirculation. J. Physiol..

[102] Jian M.Y., King J.A., Al-Mehdi A.B., Liedtke W., Townsley M.I. (2008). High vascular pressure-induced lung injury requires P450 epoxygenase-dependent activation of TRPV4. Am. J. Respir. Cell Mol. Biol..

[103] Yin J., Michalick L., Tang C., Tabuchi A., Goldenberg N., Dan Q., Awwad K., Wang L., Erfinanda L., Nouailles G. (2016). Role of Transient Receptor Potential Vanilloid 4 in Neutrophil Activation and Acute Lung Injury. Am. J. Respir. Cell Mol. Biol..

[104] Wu S., Jian M.Y., Xu Y.C., Zhou C., Al-Mehdi A.B., Liedtke W., Shin H.S., Townsley M.I. (2009). Ca^2+^ entry via alpha1G and TRPV4 channels differentially regulates surface expression of P-selectin and barrier integrity in pulmonary capillary endothelium. Am. J. Physiol. Lung Cell. Mol. Physiol..

[105] Kuebler W.M., Jordt S.E., Liedtke W.B. (2020). Urgent reconsideration of lung edema as a preventable outcome in COVID-19: Inhibition of TRPV4 represents a promising and feasible approach. Am. J. Physiol. Lung Cell. Mol. Physiol..

[106] Mole S., Harry A., Fowler A., Hotee S., Warburton J., Waite S., Beerahee M., Behm D.J., Badorrek P., Muller M. (2020). Investigating the effect of TRPV4 inhibition on pulmonary-vascular barrier permeability following segmental endotoxin challenge. Pulm. Pharmacol. Ther..

[107] Kim K.K., Kugler M.C., Wolters P.J., Robillard L., Galvez M.G., Brumwell A.N., Sheppard D., Chapman H.A. (2006). Alveolar epithelial cell mesenchymal transition develops in vivo during pulmonary fibrosis and is regulated by the extracellular matrix. Proc. Natl. Acad. Sci. USA.

[108] Rock J.R., Barkauskas C.E., Cronce M.J., Xue Y., Harris J.R., Liang J., Noble P.W., Hogan B.L. (2011). Multiple stromal populations contribute to pulmonary fibrosis without evidence for epithelial to mesenchymal transition. Proc. Natl. Acad. Sci. USA.

[109] Weber J., Rajan S., Schremmer C., Chao Y.K., Krasteva-Christ G., Kannler M., Yildirim A.O., Brosien M., Schredelseker J., Weissmann N. (2020). TRPV4 channels are essential for alveolar epithelial barrier function as protection from lung edema. JCI Insight.

[110] Bendiks L., Geiger F., Gudermann T., Feske S., Dietrich A. (2020). Store-operated Ca(2+) entry in primary murine lung fibroblasts is independent of classical transient receptor potential (TRPC) channels and contributes to cell migration. Sci. Rep..

[111] de Perrot M., Liu M., Waddell T.K., Keshavjee S. (2003). Ischemia-reperfusion-induced lung injury. Am. J. Respir. Crit. Care Med..

[112] Weissmann N., Dietrich A., Fuchs B., Kalwa H., Ay M., Dumitrascu R., Olschewski A., Storch U., Mederos y Schnitzler M., Ghofrani H.A. (2006). Classical transient receptor potential channel 6 (TRPC6) is essential for hypoxic pulmonary vasoconstriction and alveolar gas exchange. Proc. Natl. Acad. Sci. USA.

[113] Liu X., Bandyopadhyay B.C., Nakamoto T., Singh B., Liedtke W., Melvin J.E., Ambudkar I. (2006). A role for AQP5 in activation of TRPV4 by hypotonicity: Concerted involvement of AQP5 and TRPV4 in regulation of cell volume recovery. J. Biol. Chem..

[114] Sidhaye V.K., Guler A.D., Schweitzer K.S., D’Alessio F., Caterina M.J., King L.S. (2006). Transient receptor potential vanilloid 4 regulates aquaporin-5 abundance under hypotonic conditions. Proc. Natl. Acad. Sci. USA.

[115] Ma T., Fukuda N., Song Y., Matthay M.A., Verkman A.S. (2000). Lung fluid transport in aquaporin-5 knockout mice. J. Clin. Investig..

[116] Glasser S.W., Detmer E.A., Ikegami M., Na C.L., Stahlman M.T., Whitsett J.A. (2003). Pneumonitis and emphysema in sp-C gene targeted mice. J. Biol. Chem..

[117] Weissmann N., Sydykov A., Kalwa H., Storch U., Fuchs B., Mederos y Schnitzler M., Brandes R.P., Grimminger F., Meissner M., Freichel M. (2012). Activation of TRPC6 channels is essential for lung ischaemia-reperfusion induced oedema in mice. Nat. Commun..

